# A Pilot Study Exploring Caregivers’ Experiences Related to the Use of a Smart Toothbrush by Children with Autism Spectrum Disorder

**DOI:** 10.3390/children11040460

**Published:** 2024-04-11

**Authors:** Katherine France, Olivia Urquhart, Eugene Ko, Juliana Gomez, Maria Ryan, Matilde Hernandez, Marta Gabinskiy, Patricia M. Corby, Mark S. Wolff

**Affiliations:** 1 Department of Oral Medicine, School of Dental Medicine, University of Pennsylvania, Philadelphia, PA 19104, USA; kfrance@dental.upenn.edu (K.F.); patcorby@upenn.edu (P.M.C.); 2 Center for Integrative Global Oral Health, School of Dental Medicine, University of Pennsylvania, Philadelphia, PA 19104, USA; uolivia@upenn.edu; 3 Center for Clinical and Translational Research, School of Dental Medicine, University of Pennsylvania, Philadelphia, PA 19104, USA; mgabi@upenn.edu; 4 Colgate-Palmolive Company, Piscataway, NJ 08854, USA; juliana_gomez@colpal.com (J.G.); maria_ryan@colpal.com (M.R.); hernandez@colpal.com (M.H.); 5 Center Care for Persons with Disabilities, School of Dental Medicine, University of Pennsylvania, Philadelphia, PA 19104, USA

**Keywords:** autism spectrum disorders, toothbrushing, wireless technology, augmented realities, gamification

## Abstract

Background: Research on caregivers for children with intellectual disabilities, particularly those with autism spectrum disorder (ASD), has highlighted several obstacles to achieving better oral health. These include challenges with tolerating oral care, sensory processing differences, uncooperative behaviors, and communication impairments. There is limited understanding of what caregivers would consider “successful assistance” in improving oral health for these children. Objectives: This pilot study aimed to examine caregivers’ and user’s experiences with a Kids Smart Electric Toothbrush used by children with ASD. Methods: It involved open-ended interviews and questionnaires with caregivers prior to utilization of the toothbrush and after 4 weeks of product use by the child. Results: Seventeen children with ASD, aged 5–12, participated. A total of 58.8% of caregivers said their child brushed more often, and all reported brushing at least twice a day by week 4. Caregivers reported that children became more independent while brushing their teeth and achieved better quality brushing. Caregivers’ frustration with the brushing process, satisfaction with the device, and need to assist the child with brushing were improved. Caregivers did encounter some technical difficulties with the app. Conclusion: This study will assist in exploring “smart” toothbrush technologies for oral hygiene in children with ASD.

## 1. Introduction

The ASD literature consistently highlights that when guardians actively participate in the oral care routine of their child, it can foster a positive experience and effectively mitigate certain obstacles to care [1]. Little is known about what caregivers for children with intellectual disabilities would consider “successful assistance” (e.g., specific support tools and monitoring needed) for achieving better oral health. Research has identified a number of factors that contribute to poor oral health in children with ASD, including difficulty tolerating home and professional oral care, sensory processing differences, uncooperative behaviors, communication impairments, and challenges finding and accessing professional oral care services [2]. Caregivers are typically trained to provide oral hygiene to the child by performing it at the same time of day, in the same room, with the same toothbrush/toothpaste. They are typically shown a hand-over-hand technique, with the child holding the brush themselves and the caregiver’s hand over the child’s. Caregivers are instructed to brush after the child to ensure all areas are effectively cleaned. Unfortunately, many children with ASD have been aggressively opposed to routine brushing [2]. With advancements in children’s accessibility to smartphones and similar devices, the popularity and preference for smart toothbrushes have increased. A prior study highlighted that smart toothbrushes enhance motivation by stimulating interest in oral hygiene, thus refining brushing techniques [3]. Similarly, another study utilized mobile applications linked to a “smart brush” to enhance oral hygiene practices and habits. This study involved 68 children (aged 6–12 years) divided randomly into three groups. Group I received an electric toothbrush, group II received a smart toothbrush (ST), and group III used a manual toothbrush. The ST group recorded “scores” while brushing different areas of the oral cavity. Exclusively for the ST group, dentists provided feedback via the application on a weekly basis, based on brushing time, condition, and score of the teeth obtained through the application. The authors concluded that the utilization of smart toothbrushes effectively delivered dental health education and demonstrated a reduction in plaque for children aged 6–12 years [4].

ASD was first described in 1943 by Leo Kanner and consists of the following subtypes: (a) autism disorder, (b) Asperger’s syndrome, (c) Rett’s syndrome, (d) childhood disintegrative disorder, and (e) pervasive developmental disorder not otherwise specified [5]. Few studies have documented the oral health status of children with ASD [6,7,8,9], and studies where toothbrushing has been tested with this population have shown that using visual aids such as visual pedagogy, picture exchange communication systems, and video modeling systems can improve the oral hygiene of children with ASD [10,11,12,13,14]. However, little is known about whether these aids can serve as “successful assistance” for children with ASD in pursuit of better oral health as judged by their caregivers.

Smart connected technologies offer significant promise for improving the oral healthcare experience by monitoring compliance, demonstrating errors, and recording outcomes, all while gamifying the oral hygiene experience. The Colgate Kids Smart Electric Toothbrush (Smart E-Toothbrush) is a smart toothbrush that utilizes an augmented reality experience to engage children during toothbrushing through the use of games. The Smart E-Toothbrush provides guidance on brushing technique, and brushing rewards are given for successful oral hygiene performance on a cell phone/tablet application (app). In short, upon registration, the virtual reality game allows users to travel through a set of imaginary worlds to collect cartoon masks. There are five masks per world. The areas inside the mouth are divided into 16 zones, and users are guided to brush all 16 zones of their teeth. If the child brushes the correct zone and defeats the monster, he/she will gain diamonds. Users can earn up to 15 diamonds per zone. With perfect technique as brushing improves, users can collect up to 240 diamonds in one brushing session. As they improve their scores, they can purchase additional masks to try on in the photo booth window of the app and continue the game. At the end of the brushing session, users will see the stars they have won. The number of stars is decided by the number of diamonds. The more diamonds users collect, the more stars they will receive.

Studies have shown that children with autism often have specific sensory sensitivities, communication difficulties, and behavioral challenges that can impact their oral hygiene practices, dental visits, and overall oral health status [6,8,15]. This study used a “consumer approach” aimed to capture caregiver experiences, such as how easy and enjoyable the use of the toothbrush and app seemed to be for their child with ASD, as well as how useful the caregiver found the connected toothbrush. These insights will help determine the value of utilizing smart toothbrush technologies and augmented reality experiences by caregivers for the engagement of children with ASD in oral hygiene. 

Consumer research contributes to improving oral healthcare delivery, product development, and consumer engagement strategies. These studies can also be designed to assess factors influencing the choice of oral hygiene products in different populations [16]. Patient-centered care encompasses dimensions beyond clinical effectiveness and patient safety; it also prioritizes the preferences of patients as “consumers” of healthcare products and services. Therefore, incorporating “behavioral guidance” research in oral health among children with autism is essential for understanding their unique needs, preferences, and challenges related to oral hygiene and dental care. This helps identify barriers that may prevent optimal oral health outcomes in this population and informs the development of tailored interventions and strategies to improve oral health behaviors and outcomes [17]. 

## 2. Materials and Methods

### 2.1. Ethical Considerations

This study was conducted from August 2022 to November 2022 in accordance with the ethical principles of the Declaration of Helsinki and consistent with Good Clinical Practice Guidelines. Participants’ visits occurred at the University of Pennsylvania School of Dental Medicine, Center for Clinical and Translational Research. The study was registered in ClinicalTrials.gov with the number NCT05552144. IRB approval was obtained through the University of Pennsylvania prior to the start of the study (approval # 850364). Study participants and caregivers were informed about the purpose, procedures, and duration. They had the opportunity to discuss the study, and caregivers signed an informed consent form, while verbal assent was obtained from child participants. 

### 2.2. Eligibility Criteria

Study participants were children aged 5–12 years old and their responsible primary caregivers. To be included in the study, the child subjects needed to be in good general and oral health. Eligibility was determined by a review of dental and medical history and oral examination at baseline, availability for the duration of the study, and diagnosis of ASD level 1 (requiring support) or ASD level 2 (requiring substantial support). Upon oral examination, if the child presented with obvious signs of oral disease, severe dental caries, or pain as determined by the investigator, they were excluded. The oral examination focused initially on caries as part of standard care by thoroughly assessing the teeth for decay using visual inspection. Concurrently, the gums were also evaluated for visible signs of inflammation and/or bleeding upon inspection. Additionally, a comprehensive examination of oral tissues was conducted to detect any abnormalities or lesions, thus ensuring no signs of infection and/or inflammation were present in the child’s oral cavity. Children with ASD were recruited from “Kamp For Kids”, a 501c3 non-profit organization that funds and runs camps, events, and programs for children with ASD in Philadelphia, PA. Diagnosis of ASD was confirmed by reported data from the caregiver/legal representative and/or confirmed with medical records whenever available. Caregivers needed to be able to observe the child using the Smart E-Toothbrush, be available for the duration of the study, have access to either an Apple iPhone or iPad, or an Android device with access to an internet connection, be willing to download and use the app associated with the connected toothbrush, be proficient in English, and be willing to share their own and the child’s brushing experiences. 

Exclusion criteria included children undergoing active orthodontic treatment with fixed appliances, with obvious signs of oral disease, participating in another clinical study, currently using the Smart E-Toothbrush, or deemed as unsuitable for participation due to their behavior, at the investigators’ discretion. 

### 2.3. Type of Study and Design

**Figure 1:**
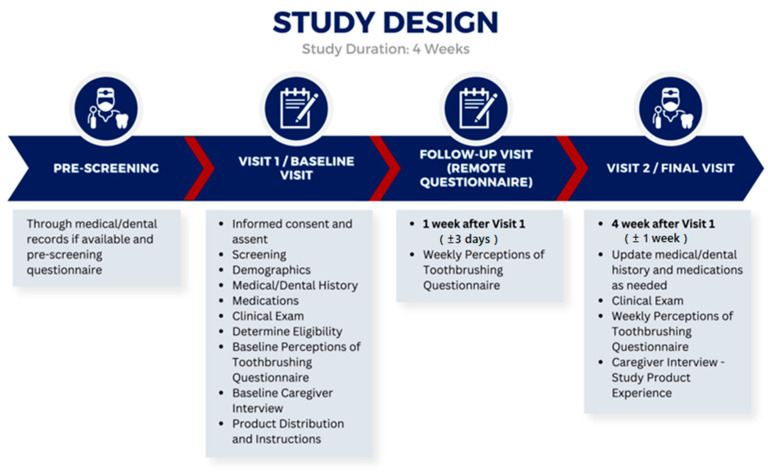
Study design: single-center, pretest–posttest, non-randomized clinical study involving interviews with caregivers prior to utilization of a Smart E-Toothbrush after 4 weeks of toothbrushing experience by the child. Questionnaires for caregivers were administered at three timepoints throughout the study, and daily text message questions about toothbrushing frequency were sent to caregivers. Interviews focused on experiences such as thoughts, feelings, intentions, observations, and behaviors upon introduction of the new device.

This was a single-center, pretest–posttest, non-randomized clinical study involving interviews with caregivers prior to utilization of the toothbrush and after 4 weeks of product use by the child; questionnaires were administered to caregivers at three timepoints throughout the study, and daily text message questions were sent to caregivers (Figure 1). 

To ensure that subjects were comfortable with the oral care regime proposed, toothbrushes were distributed based on the subject’s preference (Smart E-Toothbrush or manual toothbrush). All children opted to receive the Smart E-Toothbrush at the beginning of the study. All enrolled subjects were instructed to brush their teeth twice daily (morning and evening) for two minutes each time with the toothbrush provided. They were instructed to continue using the toothpaste they were using prior to enrollment or a regular toothpaste available for the study if they preferred. Introducing a new toothpaste flavor to the children was avoided as it could introduce bias to the brushing experience if, for example, the child did not like the taste of the new toothpaste. As part of the research protocol, at baseline, caregivers were asked to download the Smart E-Toothbrush app and create an account. They were then instructed to help their children follow the instructions on the app and brush their teeth while they played the Smart E-Toothbrush kids’ game. 

The duration of the study was 4 weeks for all subjects. All subjects were evaluated at baseline and final visits (after 4 weeks, ±1 week, of using the toothbrush). Questionnaires were conducted at baseline, 1 week (±3 days), and 4 weeks (±1 week), and interviews were conducted at baseline and after 4 weeks (±1 week) of use of the connected toothbrush and app. At baseline, week 1, and week 4, caregivers were asked to complete a questionnaire regarding the child’s brushing habits and experience with a unique link via REDCap or complete a phone/video call with the study team. REDCap is a secure, web-based, electronic data capture system hosted at the University of Pennsylvania. Interviews with the caregiver were performed to understand the range of patient experiences, such as thoughts, feelings, intentions, observations, and behaviors at baseline and related to the use of a connected toothbrush and related smartphone app after the 4 weeks of use. Data were collected through purposive sampling with in-depth semi-structured interviews. The interviews lasted between 30 and 60 min and were audio recorded. Recordings were de-identified and used for the qualitative analyses. 

Text messages containing five questions were sent twice a day (AM and PM) via TWILIO, an automated short message service (SMS) platform, for caregivers to report on the toothbrushing experiences for each day. Questions were sent to gather feedback regarding difficulty with brushing using the device, caregiver satisfaction with the brushing experience, or frustration with the experience. Questions utilized a scale (0–10), where “0” would represent, for example, “not difficult at all”, “not satisfied at all”, “not frustrated at all”, or “not helpful at all”. 

Caregivers were also asked about the frequency of their child’s brushing and could choose from three options: 0 = 0–1 Minutes; 1 = 1–2 min; 2 = More than 2 min. Child subjects were instructed to refrain from routine dental treatment during the course of the study.

### 2.4. Statistical Methods

This pilot study was conducted to examine caregivers’ and users’ experiences with a Kids Smart E-Toothbrush used by children with ASD. A priori sample size calculations for the quantitative outcomes were conducted in G*power version 3.1. With a type I error rate of 5% and 80% power, the minimum sample size needed to detect an effect size of 0.57 (2-point change on a 0–10 scale and a standard deviation for the change of 3 points) is N= 17 for a Wilcoxon signed-rank test for matched pairs. A systematic review of empirical tests for calculating sample size in qualitative research found that saturation was reached with 9–17 interviews [18]. Based on these two sources, we enrolled seventeen children with ASD, between 5 and 12 years old, and their caregivers into the study, all of whom completed the study. 

**Figure A1:**
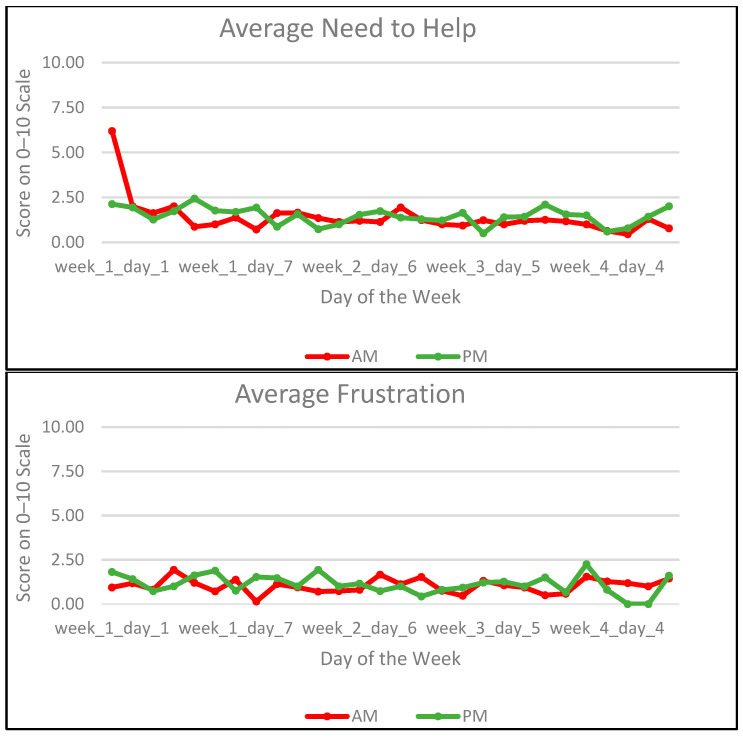

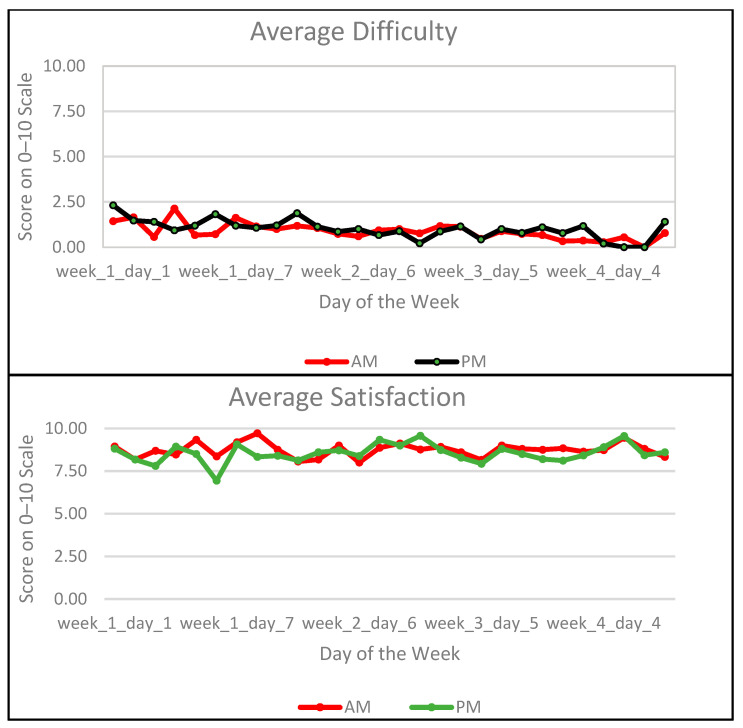
TWILIO texting data. Difficulty: on a scale of 0–10 (0 being not difficult at all and 10 being extremely difficult). Frustration: on a scale of 0–10 (0 being not frustrated at all and 10 being extremely frustrated). Help brushing: on a scale of 0–10 (0 being no help at all and 10 being a great deal of help). Satisfaction: on a scale of 0–10 (0 being not satisfied at all and 10 being extremely satisfied), in the past 7 days.

Quantitative data from the questionnaire and TWILIO: Baseline characteristics for caregivers and children (demographics, medical and dental history, and clinical findings) and adverse events were summarized with descriptive statistics. Continuous variables were summarized with means/standard deviations or medians/interquartile ranges, while categorical variables were summarized with frequencies and corresponding percentages. Two-sided non-parametric paired tests were utilized to assess changes in median scores from the answers to the perceptions of toothbrushing questionnaires between baseline and week 4. The Wilcoxon signed-rank test was implemented when the sample distributions were not heavy-tailed and when no outliers were apparent. Otherwise, the sign test was implemented. Bonferroni adjusted critical *p*-values were computed to account for multiple tests. Mean scores were calculated for the TWILIO texting data for the AM and PM questions separately, and these were plotted on line graphs (Figure A1). Data were analyzed and visualized using SAS software, Version 9.4. Copyright © 2020 SAS Institute Inc., Microsoft Corporation, Microsoft Excel 365, Version 2301, and R Statistical Software (v4.2.1; R Core Team 2022).

**Figure 2:**
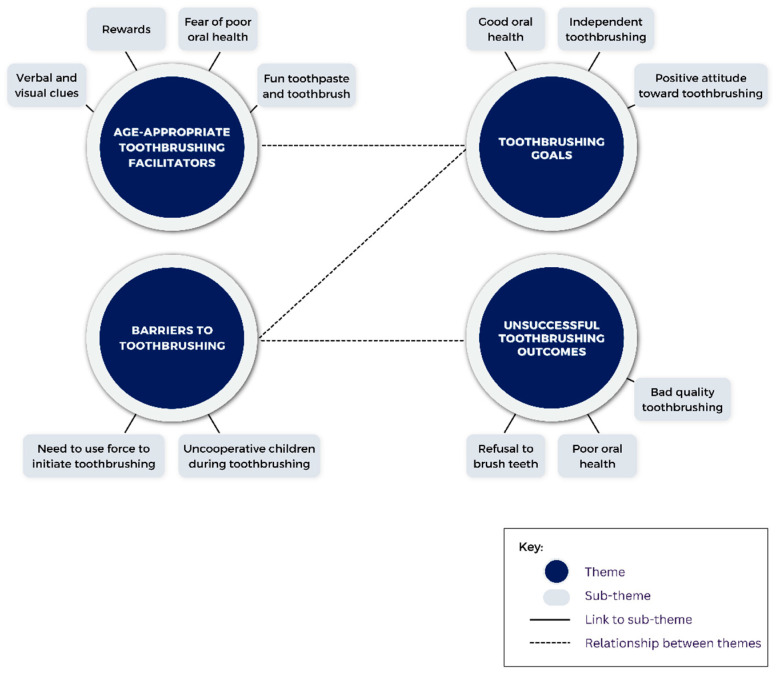
Thematic map describing themes and sub-themes emerging from baseline caregiver interviews.

**Figure 3:**
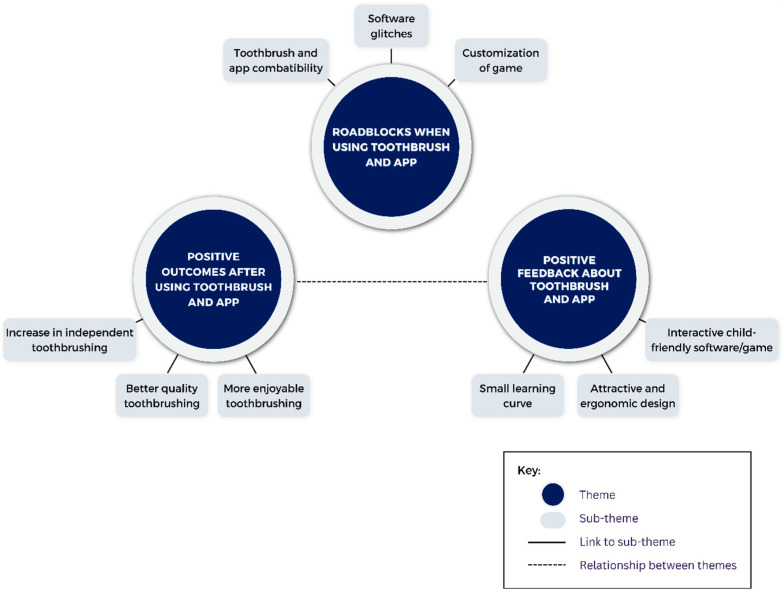
Thematic map describing themes and sub-themes emerging from caregiver interviews during the final visit.

Qualitative data from interviews: Baseline and week 4 interviews with caregivers were transcribed. Theoretical thematic analysis was conducted at a semantic level by first carrying out per-question, line-by-line coding [19,20]. Next, candidate themes and sub-themes were developed from these codes, and in an iterative process, codes, themes, and sub-themes were re-defined when necessary. From the final themes and sub-themes, we created two thematic maps, one for baseline interviews and one for follow-up interviews. (Figure 2 and Figure 3). The interviews were conducted by a trained research coordinator at a private location within the clinical research center at Penn Dental Medicine. This individual was not involved with the care of any of the research subjects prior to or during the study.

## 3. Results

### 3.1. Study Participant Demographics and Baseline Characteristics

A total of 17 participants met the study criteria and were enrolled in the study. The mean age of the child participants was 8.5 (standard deviation (SD): 2.1), and the mean age of the caregivers was 39.2 (SD: 6.5). Fifteen child participants were male, and two were female, while caregivers were predominantly female (n = 16). The majority of the participants were Black/African American and Caucasian. The caregiver was the child’s mother 88.2% of the time (Table 1). The medical and dental history of the children participating in the study was unremarkable (Table A1). 

### 3.2. Caregiver Interviews: Baseline

Baseline interviews regarding the child’s oral care routines demonstrated that no more than 35.3% of children brushed their teeth independently. One caregiver (5.9%) brushed their child’s teeth, whereas the remaining children required some help with brushing their teeth. When caregivers were questioned about their oral health routines, 70.6% brushed their teeth twice a day, 64.7% either used floss or a Waterpik to clean between their teeth, and 41.2% used mouth rinses regularly (Table A2). 

During the baseline interview with caregivers, four major themes were identified (Figure 2, Table A3):

(1) Age-appropriate toothbrushing facilitators: Caregivers elucidated a number of age-appropriate tools and tactics to facilitate toothbrushing for their children. Children may not remember to brush their teeth, so cues like setting out the toothbrush on the countertop could be useful reminders. Children are also motivated by rewards and things that look attractive or fun. Incentivizing them to brush their teeth by promising stickers or other prizes could help establish the habit until one day these are no longer needed. Tasty toothbrush flavors and bright-colored or themed toothbrushes could also be an enticing way to encourage toothbrushing (Table A3, Excerpt 1). On the other hand, instead of promoting brushing as a fun and rewarding experience, caregivers could instill the fear of getting cavities to encourage good home oral hygiene.
(2) Toothbrushing goals: During baseline interviews, caregivers listed a number of goals they wanted their children to achieve related to toothbrushing, which they hoped could be achieved with the facilitators listed above. First and foremost, a positive attitude toward brushing would be a step toward taking pride in their oral hygiene (Table A3, Excerpts 2 and 3). This pride would hopefully lead the child to take the initiative in brushing their teeth independently (Table A3, Excerpt 2). Caregivers also noted that the ultimate goal of toothbrushing is achieving and maintaining good oral health (Table A3, Excerpt 3). “*We want him to improve his oral hygiene and get more excited about brushing his teeth and this is definitely something that we are working on*”, said one caregiver while elaborating on their child’s toothbrushing goals.
(3) Barriers to toothbrushing: Caregivers encountered many barriers when toothbrushing with their kids before study initiation, including getting into arguments when it is time for brushing and having to physically force them to brush. Even during toothbrushing, the children can be uncooperative, with one caregiver lamenting that “*he would scream and yell: no I don’t want to, it hurts*” (Table A3, Excerpt 4). These barriers are roadblocks to the toothbrushing goals being achieved.
(4) Unsuccessful toothbrushing outcomes: A number of poor outcomes can result as a consequence of the identified barriers. Caregivers reported that uncooperative behavior during brushing could be one reason for poor quality and short duration of brushing. Children may also have a hard time mastering a circular motion when brushing. One caregiver noted that their child “*doesn’t do his gums very well… probably because of the circular motion… [and] needs to control movements like an adult*” (Table A3, Excerpt 5). Children may refuse to brush their teeth outright if the aforementioned barriers are not overcome. Poor quality brushing or outright refusal to brush can all lead to poor oral health, with one caregiver worried about their child “*continuing to get some kind of cavity*” (Table A3, Excerpt 6).

### 3.3. Pre–Post Questionnaires and Caregiver Interviews (Final Visit)

#### 3.3.1. Smart E-Toothbrush Use

**Figure A2:**
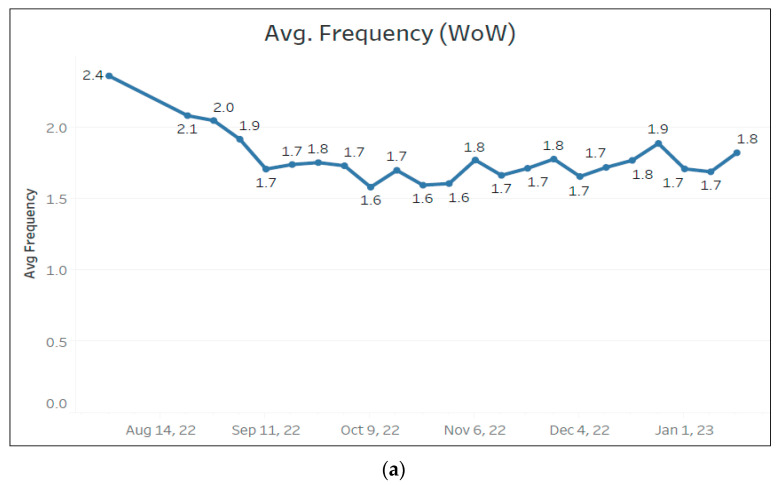

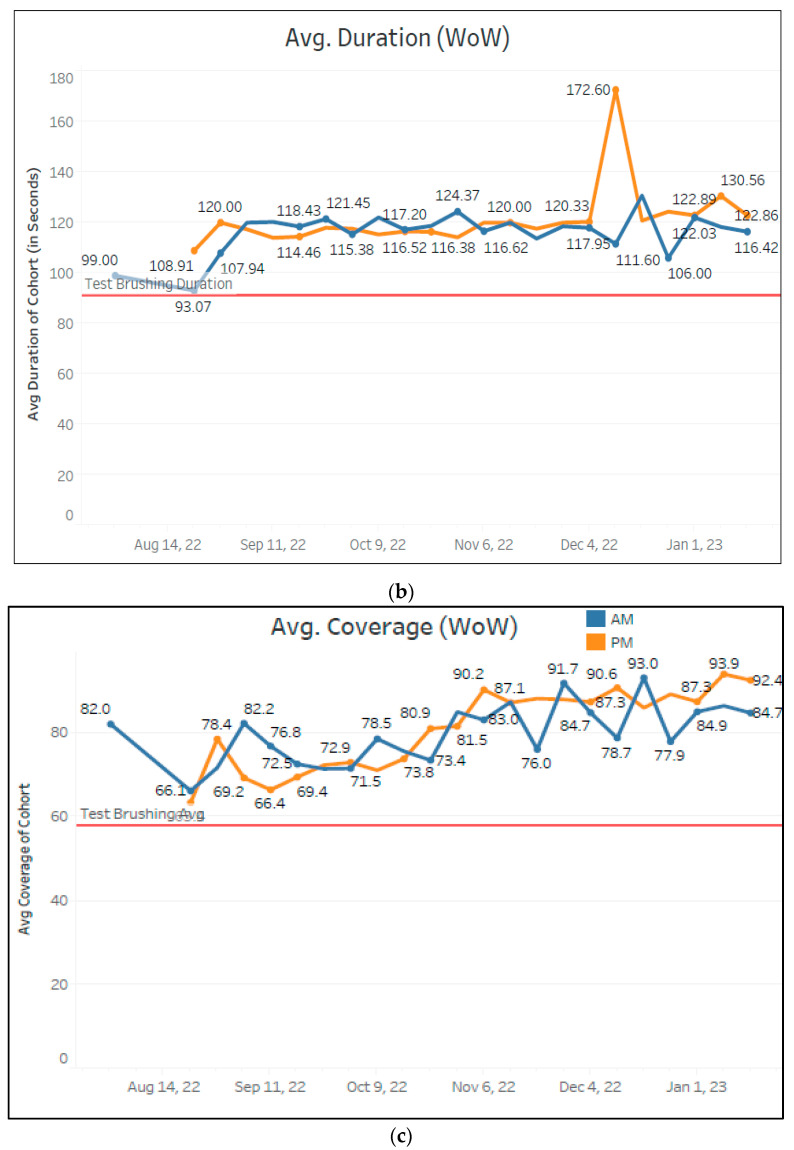
Data collected from the toothbrush sensors. (**a**) The toothbrush sensors detected an average toothbrushing frequency of 1.76 times a day throughout the course of the study. (**b**) The toothbrush sensors detected an average toothbrushing duration of 1 min and 58 s throughout the course of the study. A total of 64.7% of caregivers indicated in the week 4 questionnaire that their child brushes a little or a lot longer since using the Kids Smart E-Toothbrush. (**c**) The toothbrush sensors detected an average toothbrushing coverage of the children’s teeth of 77.2% throughout the course of the study.

Interviews with caregivers demonstrate that all children used the electric toothbrush throughout the course of the study, and 58.8% of the children allowed brushing more often (Table 2). All caregivers reported that children brushed at least twice a day (Table 2), and the data from the sensors attached to the toothbrushes were in alignment with these claims (Figure A2a). The sensors also detected an average brushing duration of 1 min and 58 s, and when caregivers were asked directly about toothbrushing duration, 64.7% indicated that their child brushes at least a little longer since starting to use the toothbrush (Figure A2b).

No adverse events were reported throughout the four-week study period.

#### 3.3.2. Smart E-Toothbrush Experience

**Figure 4:**
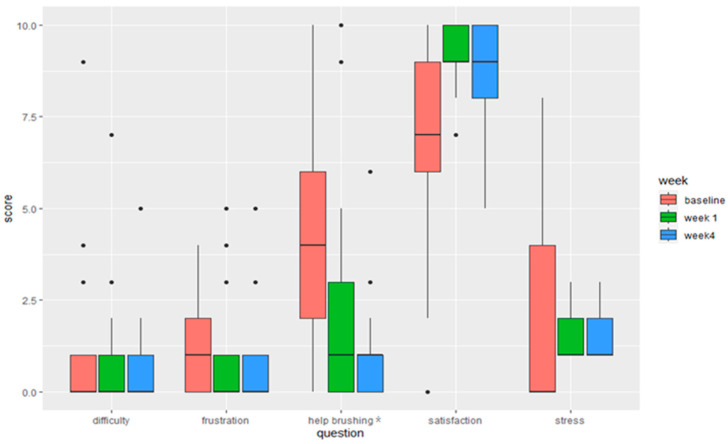
Caregivers’ experiences with the Smart E-Toothbrush from questionnaires at baseline, week 1, and week 4. Difficulty, frustration, help brushing, satisfaction, and stress: Reported on a scale of 0–10 (0 being not difficult at all, not frustrated at all, not satisfied at all, or not stressful at all and 10 being extremely difficult, extremely frustrated, a great deal of help, extremely satisfied or extremely stressful). * The change in the median response for “help brushing” was statistically significant between week 1 and week 4 with Bonferroni corrected *p*-value = 0.0041.

**Figure 5:**
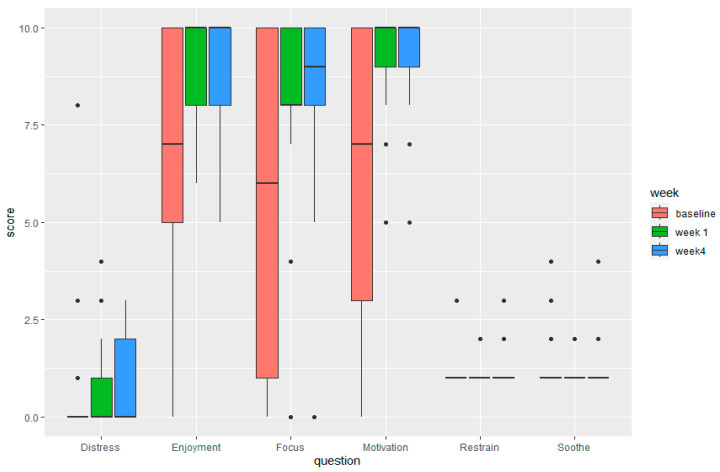
Child’s behavior when using the Smart E-Toothbrush from questionnaires at baseline, week 1, and week. 4. Distress, enjoyment, focus, motivation: measured on a scale of 0–10 (0 being not distressed, did not enjoy, not focused, not motivated, and 10 being extremely distressed, enjoyed a great deal, extremely focused, or extremely motivated). Restrain and soothe: 1= never, 2 = less than half the time, 3 = about half the time, 4 = more than half of the time, 5 = always. The dots in the figure are outliers.

**Figure 6:**
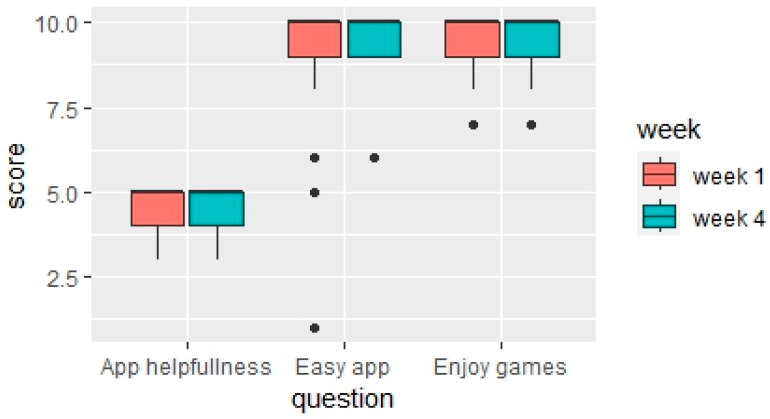
Children’s general experience with the Smart E-Toothbrush app at week 1 and week 4. App helpfulness: measured on a scale of 1–5 (1 being not helpful at all and 5 being extremely helpful). App easiness and game enjoyment: measured on a scale of 0–10 (0 being not easy at all or did not enjoy games at all and 10 being extremely easy or enjoyed the games a great deal). The dots in the figure are outliers.

**Figure 7:**
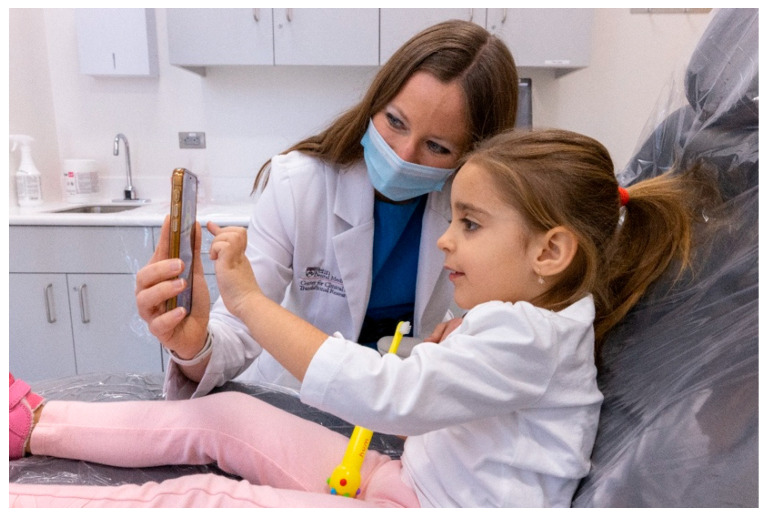
User engagement with the app.

**Figure A3:**
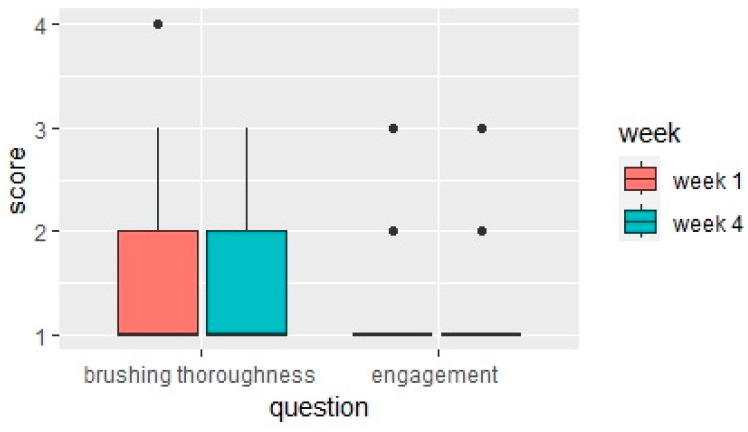
Child’s brushing thoroughness and engagement while using the Smart E-Toothbrush at week 1 and week 4. Brushing thoroughness: 1 = much improved, 2 = somewhat improved, 3 = unchanged, 4 = somewhat worse, 5 = much worse. Engagement: 1 = strongly agree, 2 = agree, 3 = neither agree nor disagree, 4 = disagree, 5 = strongly disagree.

Three themes related to the overall experience with the new smart toothbrush were identified (Figure 3, Table A3, Table A4, Table A5, Table A6 and Table A7):

(1) Positive outcomes after using the Smart E-Toothbrush and app: Caregivers reported that the quality of the toothbrushing improved after using the toothbrush for three weeks. One caregiver exclaimed that their child “*does the best job*” brushing (Table A3, Excerpt 7). This is noteworthy because, at baseline, this is something they considered a poor outcome associated with unsuccessful brushing (Figure 2). Many caregivers also reported that brushing thoroughness was much improved at weeks 1 and 4 (Figure A3), with the toothbrush sensor detecting an average toothbrushing coverage of the children’s teeth of 77.2% (Figure A2c). They also saw an uptick in independent brushing. One caregiver was proud to report that “[their child] *actually brushes his teeth solely independently*” (Table A3, Excerpt 7). This was corroborated by the quantitative questionnaires, in which a statistically significant improvement in the need for caregivers to aid their children with toothbrushing was observed (Bonferroni corrected *p*-value = 0.0041) (Figure 4 and Figure A1). Caregivers also verbalized that the children enjoyed toothbrushing more: “*He didn’t come out crying, moaning, groaning because he just had his teeth brushed*”, “*He was happy*”, and “*He achieved the goal, and he got recognition for it*”, one caregiver was happy to report (Figure 2, Table A3, Excerpt 8). Non-statistically significant improvements in the children’s enjoyment of brushing and motivation to brush were also observed from the questionnaire (Figure 5). Caregivers were also slightly less frustrated and more satisfied with the toothbrushing experience (Figure 4 and Figure A1).

(2) Positive feedback about the smart toothbrush and app: Caregivers liked the design and color of the toothbrush, noting that it was ergonomic and attractive for the kids (Table A3, Excerpt 9). “*I like the fact that it was a little bit elongated just the way it was down here*. *When he was up here brushing it wasn’t in his way*”, said one caregiver. They also noted that the kids loved how interactive the app was, including the ability to reach new levels and get rewards. They were especially happy with the instant gratification—“*He got the reward right away and that made him happy*”, one caregiver noted (Table A3, Excerpt 10). When learning how to use the app and toothbrush, they noted that, at first, it took a few tries to figure out how to get the toothbrush in the frame of the app and connect the toothbrush to the app, but once they got the hang of it, using the technology was easy. This sentiment was encapsulated by one caregiver who said, “*At first he had to know how to angle it, things like that, but after that, after he got a hang of it, it was okay*” (Table A3, Excerpt 11, Table A6 and Table A7). Overall, usage of the app was found to be easy and straightforward (Table A6). The questionnaires were in agreement with the interviews, with caregivers indicating that the app overall was extremely helpful and easy to use, and that children enjoyed the games (Figure 6 and Figure 7). Adoption of the technology was high; 100% of the children used the app, and 41.2% of the participants were excited and had fun when using the app for the first time (Table A6).
(3) Roadblocks when using the toothbrush and app:
  Some technological difficulties with the app and/or toothbrush emerged during the study. These included app connectivity issues and the app sometimes not picking up the toothbrush (Table A3, Excerpts 12 and 13), which would sometimes lead to the child’s inability to see their head in the frame of the app. One caregiver indicated that “*difficulty was with the sensor, I think might need some work*”. Children also became frustrated with the app not registering that they scored points and with game point scoring (Table A3, Excerpt 13, Table A7). Caregivers cited some suggestions for improvement. Overall, they felt that the game could have more options for customization. The app did not allow the child to choose their rewards and there was a desire to make multiple accounts for multiple children (Table A7).

## 4. Discussion

Children with special oral healthcare needs are at a greater risk for experiencing oral health disparities than the general pediatric population [21,22,23]. The result of a meta-analysis suggests that children with autism spectrum disorder tend to have poorer oral hygiene, a higher risk of caries, and a lower salivary pH compared to healthy children [24]. Dental practitioners are likely to encounter children with ASD in their practices. A recent surveillance study conducted by the Autism and Developmental Disabilities Monitoring Network (ADDM) across 11 sites in the US showed that the overall ASD prevalence per 1000 children aged 8 years was 27.6%, with one in 36 children having ASD [25]. These findings highlight the importance of dental care and oral health management in children with ASD to address the specific factors contributing to the increased risk of caries (tooth decay) and periodontal problems in children with ASD. Some of these factors were also observed in our study, and they include irregular brushing habits due to difficulties faced by trainers and parents while brushing the children’s teeth. Additionally, the side effects of medications used to manage autism symptoms, such as psychoactive drugs or anticonvulsants, may lead to generalized gingivitis in some cases, making this population highly susceptible to the development of oral diseases, including dental caries. A recent clinical trial explored the use of PT (parent training) interventions to improve dental care for children with ASD. The results of the randomized controlled trial had similar findings when compared with our study and showed that the PT intervention was effective in increasing the frequency of daily home oral hygiene and improving oral health [26]. The study also examined the feasibility, acceptability, and engagement of the PT intervention and showed similar caregiver experience, reporting that the intervention was well-received, with high retention, adherence, utilization, and satisfaction among participating families.

There are a number of behavioral and comfort factors to overcome that contribute to poor oral health in children with ASD, including sensory processing differences, uncooperative behaviors, communication impairments, and difficulties tolerating home and professional care. One study reported that as few as 50% of children with ASD brushed their teeth the recommended twice per day, and 61% of parents of children with ASD reported that toothbrushing is difficult [15]. The American Academy of Pediatric Dentistry (AAPD) recommends a number of basic behavior guidance techniques to help, including tell–show–do, voice control, nonverbal communication, and positive reinforcement. The design of a manual and battery-operated toothbrush working with an interactive app could make it easier to follow the home care guidelines recommended by AAPD [27].

Strengths of our study, limitations, and future directions

The intent of our pilot study was to further understand the perceptions of children with ASD and their caregivers when adopting the Smart E-Toothbrush and to optimize the software. 

A limitation of our methodology was the universal selection of the electronic toothbrush by all children, regardless of the choice of a manual toothbrush with gamification as an alternative. Furthermore, the small sample size contributes to the limitations of our study. Additionally, we chose not to employ randomization, a common feature of traditional case–control clinical trial designs. Consequently, our study lacked control groups. In our approach, we aimed to collect feedback from children with ASD and caregivers’ perceptions of the introduction of a novel smart toothbrush. These studies are equally important in the context of ASD as these individuals need supportive oral care from families and caregivers; thus, understanding how oral care devices can be adapted and customized to the needs of this population is important and necessary prior to the designing of large trials. We have adopted the consumer feedback approach and, working in partnership with experts, we aimed to further understand usability issues and optimization of the device and technology. 

Consumer research in the realm of oral health interventions for children with ASD is aimed at gathering insights from caregivers, healthcare providers, and individuals with ASD. This approach was also noted by Floríndez et al., who advocated for collaborative efforts between families and healthcare professionals to devise impactful strategies for oral healthcare [28]. This collaborative approach aims to mitigate the challenges associated with poor oral health among disadvantaged populations, ultimately striving to address oral health disparities among individuals with ASD [29]. The objective is to design interventions that effectively enhance access to dental care and improve the overall oral health experience for this population and their families. In the context of our study, the successful use of any device for oral care hinges on the ability to overcome challenges, such as difficulty in getting the toothbrush into the patient’s mouth. Therefore, it is imperative to first understand patient and caregiver attitudes towards the use of a toothbrush before assessing outcomes related to plaque removal or reduction in periodontal inflammation. The primary aim of this study was to develop strategies that enable the toothbrushing process for children with ASD. To accomplish our goals, most of the data analyzed were qualitative in nature, collecting caregiver-reported outcomes like quality of toothbrushing. The lack of similar oral health trials for children and adolescents with ASD on smart e-toothbrushes limited our discussion; however, most studies emphasize the importance of flexibility from parents and caregivers in supporting oral care, including utilizing in-home services for educational sessions. This highlights the necessity to address practical and logistical barriers through innovative care models, such as leveraging telehealth and oral care devices and tools for this specific population [26].

Our study suggested that children with ASD level 1 or 2 may benefit from more individualized parent training programs and interventions. Overall, our study aligns with published research that highlights the potential for these interventions to address a critical unmet healthcare need in this population. Further, community-based replication and expansion are needed to validate the findings and promote broader implementation of family education programs. 

## 5. Conclusions

This study demonstrated that caregivers reported that their children with ASD had improved motivation, focus, and enjoyment while brushing utilizing the Smart Electric Toothbrush with augmented reality. According to caregivers, the children became more independent while toothbrushing and achieved better quality brushing. Caregivers’ frustration, satisfaction, and need to help the child with brushing improved over the study duration. Overall, caregivers and children participating in the study liked the features of the application and the gamification features, using the game elements to promote user engagement with the app. 

Dental practitioners are likely to encounter children with ASD in their practices. A recent surveillance study conducted by the Autism and Developmental Disabilities Monitoring Network (ADDM) across 11 sites in the US showed that the overall ASD prevalence per 1000 children aged 8 years was 27.6%, with one in 36 children having ASD [25]. Interaction with caregivers and children regarding brushing and interdental cleaning is crucial to improve oral health and prevent oral disease in this population. Simple interventions like toothbrushing are critical for improving and/or maintaining oral health in the ASD population. Additionally, smart devices, such as the one we tested, can be implemented to improve motivation and compliance with oral care. Furthermore, subjects who become more comfortable with home oral healthcare may be more amenable to dental examination.

## Figures and Tables

**Table 1:** Child and caregiver demographics.

Demographic	Child	Caregiver
Age, years: (Mean (standard deviation))	8.5 (2.1)	39.2 (6.5)
Sex (frequency (%))		
Male	15 (88.2%)	1 (5.9%)
Female	2 (11.8%)	16 (94.1%)
Race (frequency (%))		
Black/African American	8 (47.1%)	8 (47.1%)
White/Caucasian	5 (29.4%)	7 (41.2%)
Asian	1 (5.9%)	1 (5.9%)
Native Hawaiian/Other Pacific Islander	0 (0%)	0 (0%)
American Indian/Alaska Native	0 (0%)	0 (0%)
Unknown	0 (0%)	0 (0%)
Not reported	0 (0%)	0 (0%)
Other	3 (17.7%)	1 (5.9%)
Ethnicity (frequency (%))		
Hispanic or Latino	1 (5.9%)	1 (5.88%)
Not Hispanic or Latino	16 (94.1%)	16 (94.1%)
Relationship to child (frequency (%))		
Mother	N/A	15 (88.2%)
Father	N/A	1 (5.9%)
Grandparent	N/A	0 (0.0%)
Other relative	N/A	0 (0.0%)
Non-relative	N/A	0 (0.0%)
Other (legal guardian)	N/A	1 (5.9%)

**Table 2:** Smart E-Toothbrush use during the study period.

Question	N	%
Smart electric toothbrush use during entire study period		
Yes	17	100.0%
No	0	0.0%
Frequency of smart electric toothbrush use during study		
Two times/day	15	88.2%
Three times/day	2	11.8%
Change in toothbrushing frequency		
Brush more often	10	58.8%
Brushing frequency unchanged	7	41.2%

## Data Availability

Data are available from the corresponding authors. The data are not publicly available because people (or services) might be identifiable from the interview transcript, and it would require a tremendous amount of work to ensure that all names or other information that might make an individual identifiable are redacted.

## Appendix A

**Table A1:** Child’s medical and dental history.

Variable	Frequency (%)
Child under care of a physician	
Yes	3 *** (17.65%)
No	14 (82.35%)
Child’s current physical health	
Good	16 (94.2%)
Fair	1 (5.88%)
Poor	0 (0%)
Medical conditions (current or treated for in the past) *	
Heart problems	1 (5.88%)
Epilepsy/Seizures	1 (5.88%)
Asthma or Hay fever **	5 (29.4%)
Difficulty breathing	3 (17.65%)
Skin problems	1 (5.88%)
None	9 (52.9%)
Frequency of dental visits	
Four times a year	1 (5.88%)
Twice a year	15 (88.24%)
Once a year	1 (5.88%)
History of allergies to any oral care products, personal care consumer products, or their ingredients	
Yes	0 (0%)
No	17 (100%)

* % do not add up to 100% as children can have more than one condition. ** Unable to distinguish if children previously had or currently have asthma or hay fever. *** Explanation: routine checkups, orthopedics for club foot checkups, and ENT airway department. One child has/had epilepsy/seizures, asthma or hay fever, and difficulty breathing.

**Table A2:** Child and caregiver oral healthcare routines at baseline.

Child’s Oral Care Routines		Caregiver’s Oral Care Routines	
Question	Frequency (%)	Question	Frequency (%)
Does your child use toothpaste?		Tell me about your typical oral care routines *	
Yes	17 (100%)	Brush teeth twice a day	12 (70.6%)
No	0 (0%)	Interdental cleaning (floss, Waterpik)	11 (64.7%)
What is the name of toothpaste?		Mouthrinse	7 (41.2%)
Colgate	4 (23.5%)	Hygiene visits (once or twice a year)	2 (11.8%)
Colgate Kids	6 (35.3%)	Caregiver does not have a routine	2 (11.8%)
Crest Kids	3 (17.6%)		
Kids with fluoride	1 (5.9%)	How do you think your oral care routines impact your child?	
ACT	1 (5.9%)	Child motivated to brush teeth by observing caregiver/sibling	8 (47.1%)
Charcoal	1 (5.9%)	Child and caregiver brush teeth together	3 (17.6%)
Colgate baking soda with peroxide	1 (5.9%)	No impact because child does not observe caregiver brushing teeth	3 (17.6%)
What is the flavor? *		Unclear/did not answer question	3 (17.6%)
Bubblegum	11 (64.7%)		
Spearmint	3 (17.6%)		
Fruity	2 (11.8%)		
Strawberry	1 (5.9%)		
Peppermint	1 (5.9%)		
Child’s typical toothbrushing routine **			
Child brushes teeth independently (*Answers to two questions overlapped and are presented together*)	5 (29.4%)/6 (35.3%)		
Child requires some help brushing (caregiver checks teeth to ensure they are brushed properly and either verbally informs child or helps child brush teeth, requires prompting) (*Answers to two questions overlapped and are presented together*)	5 (35.3%)/8 (47.1%)		
Caregiver and child brush teeth together	1 (5.9%)		

* % do not add up to 100% as respondents were able to choose/indicate more than one option. ** Other aspects of the children’s routine included brushing teeth twice daily (N = 4), interdental cleaning (e.g., floss, Waterpik) (N = 3), caregiver preparation of toothbrush (N = 3), visual or audible prompts before or during brushing (e.g., app, timer) (N = 3), mouth rinsing (N = 2), use of a manual toothbrush (N = 1), and caregiver brushing the child’s teeth (N = 1).

**Table A3:** Excerpts from caregiver baseline and follow-up interviews.

Number	Excerpt
1	“He likes the flavor of the toothpaste, the timer helps, likes prizes when he goes to the dentist, if you have a good report, she gives you a coin and you get to pick a prize, yea, so that’s a little incentive for him.”
2	“I really wish that he wouldn’t need my help at all. That would be great. I wish that he would may be like it more, if he likes it, he would be into doing it more by himself and enthusiastic to do it, just makes it teaching him, you know, building those habits would be easier.”
3	“We want him to improve his oral hygiene and get more excited about brushing his teeth and this is definitely something that we are working on.”
4	“He would scream and yell: no I don’t want to, it hurts.”
5	“…he doesn’t do his gums very well, so yea, it’s probably because of the circular motion, that he can’t really, I mean, he does it a little bit, but he’s not like how I would do it. He needs to learn to control movements like an adult.”
6	“…not doing his brushing and continuing to get some kind of cavity.”
7	“It’s been great, he actually brushes his teeth solely independently and he does the best job.”
8	“He didn’t come out crying, moaning, groaning because he just had his teeth brushed. He was happy. He achieved the goal and he got recognition for it. That made a big difference in his teeth. And he went to school and told everybody. About his toothbrush, the app.”
9	“He is very into feeling and texture and things like that and that was one thing; it was comfortable in his mouth. And I think he like the buzzing. You know he had those little spin brushes and stuff before and he liked it. But he grew tired of it. I like the fact that it was a little bit elongated just the way it was down here. When he was up here brushing it wasn’t in his way.”
10	“I was just happy that he was engaged and he got a reward. It was an instant reward. It wasn’t you do all this and we go do something. He didn’t have to wait. He got the reward right away and so that made him happy.”
11	“At first he had to know how to angle it, things like that, but after that, after he got a hang of it, it was okay.”
12	“I think the directions are really easy for kids, but the difficulty was with the sensor, I think might need some work, the sensors on the toothbrush.”
13	“Having to keep his head in the frame. To interact. That was the hardest part because, um, you would set it up and he would be brushing. It wasn’t really moving around. It was just that … we had to keep moving him. Its like it would drift. So I mean. Here is the screen and the space for his head is this tiny oval. If that actual thing is wider, he would be so much easier because then you don’t have to keep redirecting.”

**Table A4:** Benefits of using the Smart E-Toothbrush from the caregiver perspective.

Benefit	N (%)
Breath smells better	17 (100.0%)
Teeth look better	16 (94.1%)
Enjoys brushing more	17 (100.0%)
Improved brushing skills	17 (100.0%)
Does a better job of brushing	17 (100.0%)
Increased motivation to brush	17 (100.0%)
Pays more attention when brushing	16 (94.1%)
More focused on brushing	17 (100.0%)
Brushes for a longer amount of time	15 (88.2%)
Spends more time brushing each area of the mouth	17 (100.0%)

**Table A5:** Child’s experience with the game and app features based on caregiver interviews at week 4.

Question	Frequency (%)
How did your child react to the game?	
Positive (excitement, likes/loves it, laughed the whole time, motivator—child wants to brush teeth again, liked the rewards—getting enough diamonds for new mask/skins, picking different levels, loves the character)	16 (94.1%)
Neutral (a little fun)	1 (5.9%)
What did your child think of the characters (Dr. Molar, Karios, cavity monsters)?	
Indifferent/didn’t pay much attention to them	4 (23.5%)
Positive general (liked them, thought they were fun or cool, liked how they would get rid of germs)	13 (76.5%)
Did you take pictures when using the app?	
Yes	13 (76.5%)
What was that like? *	
Positive general (liked it, interesting, fun, loves to take pictures, enjoyable)	10 (77.0%)
Positive specific (liked the masks, faces, and hair)	4 (30.8%)
Neutral (fine)	1 (7.7%)
Negative (didn’t like getting stuck on same filters)	1 (7.7%)
No	4 (23.5%)
Did your child enjoy taking pictures? **	
Yes	15 (100%)
Did your child like using the toothbrush and the game?	
Yes	17 (100%)
What did he/she like about it?	
Games (music, characters, points and prizes, seeing themselves on the screen, taking pictures, enticed child to brush teeth)	16 (94.1%)
Toothbrush	1 (5.9%)
What did he/she dislike about it? *	
Nothing	12 (70.6%)
A lot of spit/drool until they figured out the pause/spit control button	2 (11.8%)
Did not answer question/Unclear	3 (17.6%)
Lower scores on the game even when child brushed everywhere	1 (5.9%)

* Percentages do not add up to 100% as caregivers could have cited more than one category. ** A total of 2 caregivers were not asked this question; the denominator is 15.

**Table A6:** Caregiver’s experience connecting and using the app.

Question	Frequency (%)
What was it like when you had to set up the app? *	
Easy, straightforward, simple	15 (88.2%)
Fine/Alright	1 (5.9%)
Caregiver did not set up app	1 (5.9%)
How about when you had to connect it to the toothbrush?	
No problems/easy	15 (88.2%)
Alright, some technical difficulties	1 (5.9%)
Sometimes the app didn’t read the toothbrush	1 (5.9%)
What was it like when your child used the connected toothbrush to brush his/her teeth for the first time? **	
Child was excited and had fun	7 (41.2%)
It was an easy experience	5 (29.4%)
Took a little longer to figure out compared to rest of the times	3 (17.6%)
The child liked the experience	3 (17.6%)
Child did not encounter any issues	1 (5.9%)
Did your child use the app?	
Yes	17 (100%)
How often did your child use the app?	
Twice a day	15 (88.2%)
Three times a day	2 (11.8%)

* The research team set up the app at baseline. ** Percentage does not add up to 100% as caregivers could have cited more than one category.

**Table A7:** Caregiver’s experience with the Smart E-Toothbrush based on caregiver interviews at week 4.

Caregiver’s Experience with the Smart E-Toothbrush			
Questions about the Toothbrush		Questions about the App	
What do you like about the toothbrush? *	Frequency (%)	What do you like about the smartphone app?	Frequency (%)
Interactive (comes with the app)	10 (77.0%)	Easy to download and use	6 (35.3%)
Makes toothbrushing easier	3 (17.6%)	Overall positive experience (non-specific feedback)	3 (17.6%)
Visually appealing (e.g., color)	2 (11.8%)	Games	3 (17.6%)
Increases independence	3 (17.6%)	Ensures good quality brushing	2 (11.8%)
Easy to use	2 (11.8%)	Motivates child to brush teeth	2 (11.8%)
Takes stress off parents	2 (11.8%)	Parent can track progress via email	1 (5.9%)
Child is brushing teeth more often	1 (5.9%)	What do you dislike about the smartphone app? *	
Functional (elongated shape of the toothbrush facilitates easier brushing)	1 (5.9%)	Nothing	8 (47.1%)
Different levels of vibration	1 (5.9%)	Connectivity issues/not registering the toothbrush in the frame	5 (29.4%)
What do you dislike about the toothbrush?		Inability to make multiple accounts	1 (5.9%)
Nothing	8 (47.5%)	Inability to choose mask	1 (5.9%)
Occasional connectivity issues (app not recognizing the toothbrush, having to keep head in the frame)	5 (29.4%)	Drains battery of device	1 (5.9%)
Toothbrush form (too bottom heavy, bristles too small for big kids, too long to fit in the screen for the app, no case)	2 (11.8%)	App freezes	1 (5.9%)
Issues tracking proper coverage while brushing	1 (5.9%)		
Inability to change the timer if child is slower at brushing	1 (5.9%)		

* Percentages do not add up to 100% as caregivers could have cited more than one category.
